# Workplace Chinese training in a Chinese-managed factory in Morocco: a transfer-sensitive CIPP evaluation

**DOI:** 10.3389/fpsyg.2026.1835254

**Published:** 2026-05-14

**Authors:** Yushuang Zhang, Zhaohui Huang, Qianru Gao

**Affiliations:** Beijing Polytechnic University, Beijing, China

**Keywords:** CIPP evaluation, learning persistence, training transfer, vocational language education, workplace Chinese training

## Abstract

**Introduction:**

In overseas Chinese-managed manufacturing settings, local employees' demand for Chinese is shaped by job tasks and cross-cultural coordination. This study examines workplace Chinese training in a Chinese-managed factory in Morocco.

**Methods:**

Data came from three stage-specific survey waves: pre-course needs survey (*n* = 70), mid-course process survey (*n* = 22), post-course outcome survey (*n* = 7), open-ended feedback (*n* = 8), one teacher interview, and attendance and performance records (40 valid score records). An improvement-oriented Context-Input Process-Product (CIPP) framework and training-transfer logic guided descriptive statistics and qualitative thematic analysis.

**Results:**

Findings show that learner demand was strongly task- and scenario-oriented, and materials and classroom climate were generally evaluated positively. Pacing remained the main challenge for beginners. Attendance was strongly associated with composite performance (Spearman's ρ = 0.90, *p* < 0.001) and remained substantial after removing the attendance component (ρ = 0.78, *p* < 0.001). The evidence did not directly demonstrate workplace transfer but indicated transfer-related conditions, including confidence growth, task-based rehearsal, recoverability after absence, peer support, and organizational time protection.

**Discussion:**

Workplace Chinese training in production-constrained settings should be evaluated not only by satisfaction or test performance, but also by conditions that sustain participation and enable later workplace use. The findings support a cautious, improvement-oriented interpretation of training effectiveness in a single-site industrial context.

## 1 Introduction

As Chinese enterprises expand globally, language competence in overseas factories is increasingly tied to productivity, safety, and coordination. In factory environments, pre-shift briefings, quality reporting, equipment maintenance, and procurement communication all occur under time pressure. Misunderstood information can therefore produce not only inefficiency, but also safety and quality risks. For local employees in overseas workplaces, learning Chinese is often less a long-term investment in general proficiency than an immediate resource for getting work done. Compared with school-based language learning, workplace language learning is shaped by production rhythms, shift systems, and performance pressure. Evaluating such programs requires attention not only to satisfaction or test scores, but also to whether participation can be sustained and whether learners begin to show signs of later workplace use (Baldwin and Ford, 1988; Ford et al., 2018; Hughes et al., 2020).

Research on Chinese for Specific and Professional Purposes (CSP/CVP) emphasizes the value of needs analysis and occupational alignment (Chan, 2019; Jung, 2016). In this article, workplace Chinese training is used as the main descriptor for language learning embedded in actual enterprise collaboration. Related terms such as vocational Chinese, business Chinese, and CSP/CVP are relevant, but they are treated here as adjacent labels rather than interchangeable primary concepts. Existing accounts of enterprise language programs often describe implementation experiences or summarize outcomes, yet they say less about how needs, course design, participation conditions, and transfer-related indicators can be interpreted together in an improvement-oriented evaluation.

This omission matters because participation in training is not equivalent to workplace use. Transfer research shows that learning does not automatically become behavior, and that training design and the work environment jointly shape whether transfer is attempted, sustained, or avoided (Ford et al., 2011; Huang et al., 2015). For workplace Chinese training, being able to perform in class does not mean being willing or able to use Chinese under production pressure. An analytically useful evaluation therefore needs to distinguish between direct evidence of workplace transfer and earlier indicators or conditions that make later use more or less plausible.

Generative AI provides an additional methodological question. Large language models can help organize short qualitative responses and synthesize candidate themes, but they can also introduce anchoring bias and overconfident interpretation, especially in small and heterogeneous datasets (Messeri and Crockett, 2024; Schroeder et al., 2025). In the present study, LLM assistance was treated as a bounded methodological aid rather than a substantive focus: model output was limited to candidate organization and was always subject to researcher review and an audit trail (Barros et al., 2025; Pattyn, 2024).

Against this background, the present study examines the Center for Chinese Language and Professional Skills (CCLPS), a workplace Chinese program implemented in a Chinese-managed factory in Morocco. Using an improvement-oriented, single-site mixed-methods case-study design, the study organizes evidence through the dual lens of the CIPP framework (Context-Input-Process-Product) and training-transfer logic. It addresses four research questions: RQ1 What core learning needs emerge in relation to workplace tasks, terminology, and scenarios? RQ2 Which contextual and process factors appear to support or hinder sustained participation? RQ3 What transfer-related indicators and constraints emerge from post-course, interview, and performance evidence? RQ4 What priorities for subsequent course revision can be derived from the combined evidence? The article makes three contributions. First, it applies a transfer-sensitive CIPP framework to workplace Chinese training in an overseas factory setting. Second, it highlights participation continuity and recoverability after absence as central process conditions linking course design to product-level indicators. Third, it documents a bounded human-in-the-loop workflow for using LLMs in small-scale qualitative support without ceding final interpretation to the model.

## 2 Literature review

### 2.1 Workplace language education and transfer of training

Transfer-of-training research has long argued that a gap often exists between learning outcomes and workplace behavior, and that successful transfer depends on three broad sets of factors: learner characteristics, training design, and the work environment (Baldwin and Ford, 1988). This proposition applies directly to workplace language education. Even when learners acquire relevant linguistic resources, those resources may not become action if opportunities for use are scarce, the perceived cost of error is high, or the organizational climate does not encourage experimentation. Ford et al. (2011), in their critique of the “10% transfer” myth, caution evaluators against treating participation in training as a proxy for behavioral change (Ford et al., 2011). Instead, transfer conditions themselves must be incorporated into both program design and program evaluation.

Subsequent research has refined this insight by distinguishing between “maximum transfer” and “typical transfer.” Maximum transfer refers to what learners can do under optimal conditions, whereas typical transfer refers to what they actually do in routine work settings (Huang et al., 2015). These two forms of transfer may have different predictors. In workplace language education, classroom tests and oral demonstrations are more closely aligned with maximum transfer, while attendance, work pressure, peer assistance, and real task opportunities are more likely to illuminate typical transfer. This distinction is consequential. A course may appear effective if judged only by classroom performance, yet still fail to generate regular use on site. Evaluations that privilege maximum transfer alone may therefore misread the real workplace value of a language program.

Methodologically, transfer evaluation requires multiple sources of evidence and multiple layers of indicators. Kraiger et al. (1993) proposed distinguishing cognitive, skill-based, and affective learning outcomes, which is useful for separating knowledge acquisition, operational performance, and self-efficacy or attitudinal change (Kraiger et al., 1993). In a language project, skill indicators may map onto task completion, affective indicators may reflect speaking confidence and anxiety, and environmental indicators may capture opportunity structures and support intensity. Hughes et al. (2020), in a meta-analysis of training sustainment, show that the work environment has a significant influence on whether training effects are maintained. In enterprise settings, organizational support should therefore be treated as a central explanatory variable rather than dismissed as background noise (Hughes et al., 2020).

These insights are especially relevant for adult beginners in workplace Chinese programs. The challenge is not simply whether learners have encountered target vocabulary or sentence patterns, but whether they are willing and able to mobilize those resources in consequential workplace interactions. This makes transfer a better conceptual lens than achievement alone. It also suggests that evaluation systems should avoid equating performance with proficiency in a narrow sense. What matters is the capacity to use language under real constraints, in interaction with real people, for the completion of real tasks.

### 2.2 Course design for vocational language education

Vocational language courses generally begin with needs analysis and then decompose target-language demands into executable tasks, communicative scenarios, and genres (Jung, 2016). Task-based language teaching (TBLT) places authentic or near-authentic communicative tasks at the center of instruction and supports learners' development of usable language through meaning negotiation and output (Ellis, 2003). In occupational settings, tasks are frequently tied to process language, specialized terminology, role-based scripts, and response strategies. Effective instructional design must therefore address not only language forms but also task procedures and collaboration norms.

From a competence-based perspective, course objectives should also be aligned with job competence and articulated in terms of observable performance rather than abstract content coverage (Weinert, 2001). For vocational Chinese, this suggests a teaching architecture organized around task sequence, terminology domain, and interaction strategy. A practical sequence is to establish a reusable framework for high-frequency collaborative events first, and then extend terminology and genres by functional area or role. Such a design can balance immediate usability with longer-term systematization. Learners begin with expressions that enable them to ask, clarify, confirm, report, and request support, and only later broaden into more specialized language.

It is important, however, not to confuse “authentic task” with the direct relocation of classroom instruction into the workshop. Real workplace tasks are often high-stakes and high-risk. For beginners, immediate use of the target language in live production contexts may increase anxiety and reinforce avoidance if errors are penalized socially or functionally. Course design therefore needs an intermediate layer that is simulated, low-risk, and repeatable. Scenario simulation, role-play, scripted dialogue, and immediate feedback allow learners to build expression resources in a safer environment before moving toward workplace micro-tasks. This staged pathway helps explain why learners in the present study expressed such strong preference for scenario-based activities.

The implication is that task authenticity should be calibrated rather than absolutized. What matters is not maximal realism at any cost, but a developmental gradient from rehearsal to use. In this respect, effective vocational language design is less about importing workplace pressure into the classroom than about building bridge structures between classroom learning and workplace participation. This bridging function becomes particularly important when learners have limited attendance and need instructional experiences that are both efficient and recoverable.

### 2.3 Learning persistence

Adult learners' sustained participation is shaped by the interaction of instrumental goals, self-efficacy, classroom experience, and opportunity cost (Csizér, 2019). When learners experience success in real communication, self-efficacy increases and participation tends to deepen. Conversely, absence, broken pacing, and heavy workloads can reduce the sense of control and prompt withdrawal. In workplace settings, learners often evaluate study not only in terms of intrinsic interest but also in terms of whether its return is visible and whether the investment is worthwhile. Courses that fail to provide early signs of usable progress may struggle to sustain engagement even when learners initially report positive intentions.

This dynamic is amplified by organizational conditions. When learning time is repeatedly compressed, and absences are not compensated for by recovery mechanisms, individual effort has difficulty turning into stable progress. Persistence is therefore not a purely personal trait. It is partly designed, partly supported, and partly governed. For workplace Chinese programs, this means that maintaining participation requires more than motivating content. It requires visible short-term gains, manageable task demands, and concrete structures that allow learners to re-enter the learning trajectory after disruption.

The literature on adult and workplace learning also suggests that persistence is relational. Supportive peers, clear expectations, low-anxiety environments, and accessible follow-up resources help sustain effort over time. In this respect, persistence should be understood as an emergent property of the learning ecology rather than simply the outcome of individual willpower. Programs that seek durable participation need to design for continuity, not merely for initial uptake.

### 2.4 Evaluation framework

The CIPP model organizes evaluation into Context, Input, Process, and Product, with the aim of supporting improvement-oriented decision making (Stufflebeam and Zhang, 2017). In the present study, CIPP is used primarily as an analytic framework for organizing and interpreting evidence, rather than to claim that a complete intervention cycle has already been fully implemented and systematically validated. It provides a coherent evaluative structure through which needs, design, implementation conditions, and product-level evidence can be examined in relation to one another.

When considered together with transfer logic, CIPP helps distinguish product-level indicators from the contextual and implementation conditions through which they emerge. Were needs identified with sufficient accuracy? Were resources and task designs aligned with those needs? What barriers to participation arose during implementation? What product-level indicators became visible, and how should they be interpreted in light of the limits of the available evidence? For workplace Chinese training, this combined framework enables a more cautious and decision-relevant interpretation of evidence than reliance on end-point outcomes alone.

### 2.5 Usability and risk governance of generative AI in qualitative research

Recent scholarship indicates that LLMs can assist several stages of qualitative inquiry, including coding suggestions, thematic summarization, cross-text alignment, and structured reporting (Castellanos et al., 2025; Gao et al., 2023; Zhang et al., 2025). These affordances are attractive in small research teams or fast decision contexts because they can reduce repetitive analytic labor. Yet the risks are equally clear. Models may generate plausible interpretations that are insufficiently grounded in the data, or prompt wording may anchor the direction of analysis and subtly draw researchers toward model-shaped categories (Nguyen and Welch, 2026; Schroeder et al., 2025).

Methodological reviews have accordingly proposed a set of governance principles: use only the minimum necessary input, remove identifying information, explicitly instruct the model not to fabricate, require structured outputs, maintain researcher review, and preserve an audit trail (Barros et al., 2025; Pattyn, 2024). More broadly, warnings about generative AI and “illusions of understanding” underscore the need for epistemic caution, especially in small-sample and high-uncertainty settings (Messeri and Crockett, 2024; Salah et al., 2024). In the present study, LLMs are therefore positioned not as autonomous interpreters but as controlled tools for organizing candidate evidence and supporting analytic consistency. Transparency and auditability are treated as methodological requirements rather than optional supplements.

### 2.6 Learning communities and social support

Communities of Practice (CoP) theory emphasizes that learning is not merely an internal cognitive process but a gradual formation of participation through shared practice, collaboration, and identity negotiation (Wenger, 1999). In workplace language education, language is not simply an object of study; it is also a resource for action. Learners need repeated opportunities to try, receive feedback, and adjust their communicative strategies within authentic or quasi-authentic interaction. For adult beginners, peer assistance can substantially reduce the cost of speaking by ensuring that someone is available to practice with, provide corrections, and share resources such as glossaries, template expressions, and scenario scripts.

Lave and Wenger's (1991) notion of legitimate peripheral participation is also instructive here. Learners often enter a practice through low-risk tasks before moving into more consequential participation (Lave and Wenger, 1991). In a vocational Chinese program, this may mean beginning with classroom or group-based rehearsal, then moving toward workplace micro-tasks. From this perspective, a learning community is not an accessory to formal instruction. It is infrastructure that connects classroom learning to site-based use.

Taken together, the literature suggests three linked evaluation priorities for workplace Chinese training: alignment between workplace tasks and course content, continuity of participation under production constraints, and transfer-related indicators that remain clearly distinguished from direct workplace transfer. CIPP provides the improvement-oriented scaffold for organizing these dimensions, while transfer theory and CoP explain why process conditions and opportunity structures matter. These linked priorities guide the analysis that follows.

## 3 Project context

### 3.1 Setting and program overview

The study was conducted in a Chinese-managed manufacturing factory in Morocco. The company offered a workplace Chinese program, the Center for Chinese Language and Professional Skills (CCLPS), to local employees. The program aimed to improve basic communicative capacity with Chinese colleagues and managers in production collaboration and to provide language support for business functions such as quality management, equipment coordination, and procurement communication. Instruction was delivered primarily face to face, supplemented by after-class materials and exercises. Participants were incumbent employees who enrolled voluntarily and represented a range of departments, including production, quality, and logistics. The setting thus reflects a typical profile of adult learners with no prior Chinese background and strong work-related constraints on learning.

### 3.2 Evaluation and course-operation data structure

Program performance data were drawn from internal company records and included attendance, classroom performance, assignments, and tests. These components were weighted into a composite score: attendance 10%, classroom performance 25%, assignments 10%, and tests/examinations 55%. This structure reflects a common enterprise-training logic that values both process participation and outcome performance. On the one hand, the system emphasizes learning persistence through attendance and assignments; on the other, it captures degree of mastery through formal assessment. It should be noted, however, that classroom performance scores in this cycle were almost uniformly full marks and therefore had limited discriminating power. For this reason, composite scores are reported primarily as descriptive indicators, and any association between attendance and performance is interpreted cautiously, including a sensitivity check that removes the directly embedded attendance component.

### 3.3 Learner sample

**Figure 1:**
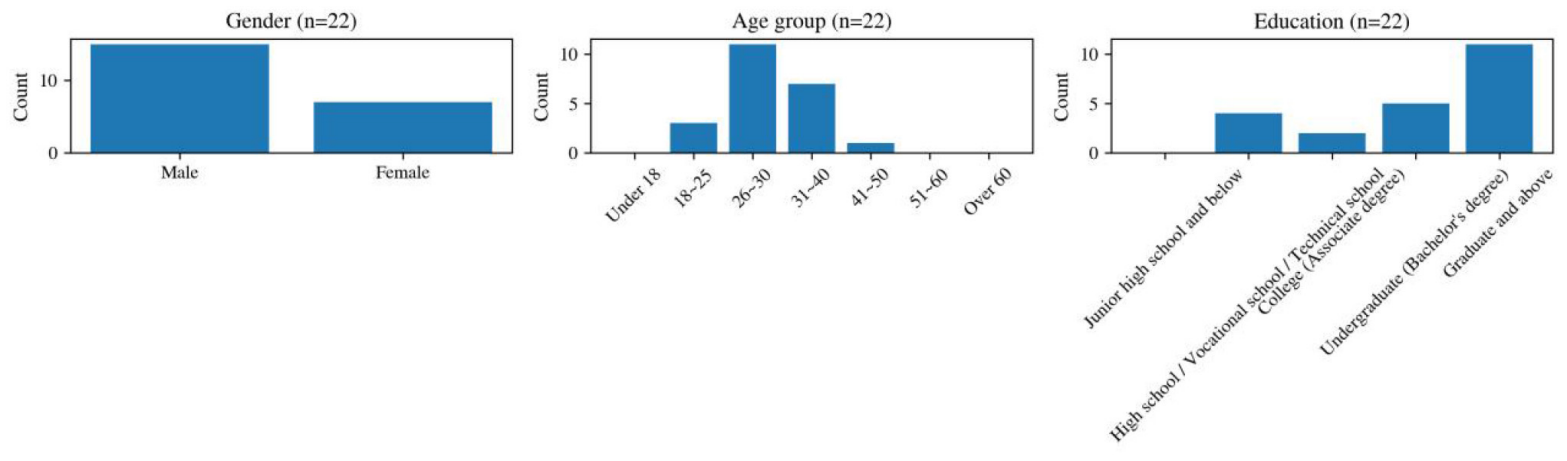
Demographic profile of the mid-course questionnaire sample (*n* = 22).

The analytic sample for the mid-course process questionnaire comprised 22 learners, including 15 men (68.18%) and 7 women (31.82%). As shown in Figure 1, the age structure was concentrated in the 26–40 range, which accounted for 81.82% of respondents. In terms of educational background, 50.00% reported postgraduate education, 22.73% undergraduate education, 18.18% high school or technical secondary education, and 9.09% junior college education. Most respondents had no prior experience learning Chinese, with 17 learners (77.27%) indicating that they were complete beginners.

The post-course outcome questionnaire yielded 7 valid responses, while open-ended feedback was available from eight texts. In addition, valid performance records suitable for score-based analysis were obtained for 40 learners. Of the 52 registered learners, the remaining 12 were trial or experience participants and were therefore excluded from the final score analysis. These datasets should be understood as stage-specific analytic subsets rather than as a matched longitudinal cohort.

### 3.4 Features of instructional implementation

The teacher interview provided contextual evidence about implementation conditions. The instructor emphasized three points. First, because most learners were beginners, the course relied heavily on repeated practice to build a usable foundation. Second, reduced contact hours and work pressure limited practice time, making it difficult to balance explanation and output. Third, after-class review, homework submission, and stable attendance were hard to sustain because of workload, personnel mobility, and shift changes. The teacher also stressed that a relaxed classroom climate and more opportunities to use Chinese were important for maintaining participation.

## 4 Research design and methods

### 4.1 Research paradigm

**Figure 2:**
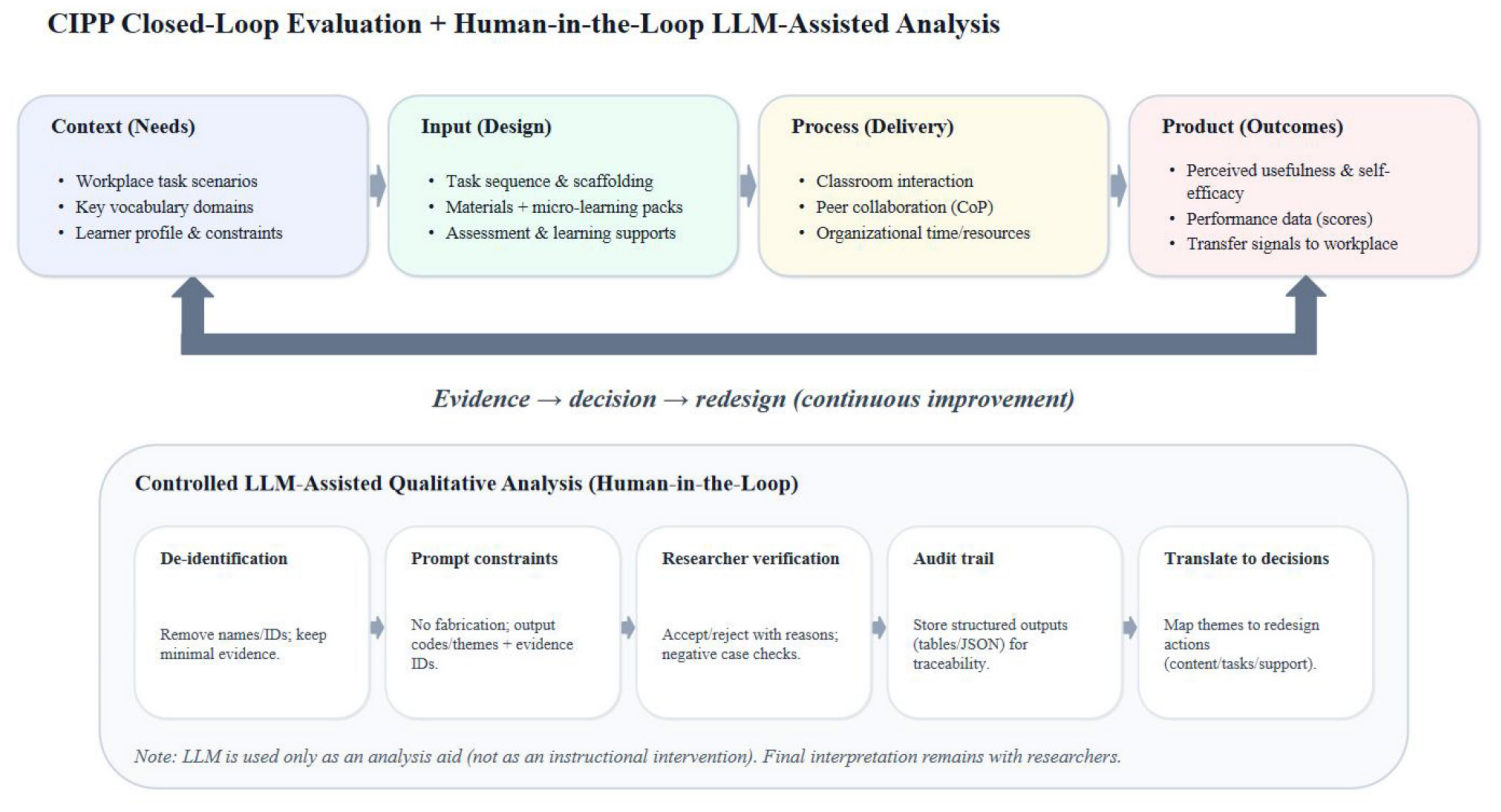
Transfer-sensitive CIPP evaluation and bounded LLM-assisted thematic-analysis workflow.

This study adopted an explanatory mixed-methods design within a single-site exploratory case-study framework. Quantitative evidence from surveys and performance records was first used to describe response distributions, learning tendencies, and bounded associations among key variables. Qualitative thematic analysis was then employed to interpret the mechanisms, constraints, and contextual conditions underlying these quantitative patterns. The two strands were subsequently integrated to generate evidence-based priorities for course revision and improvement (Creswell and Clark, 2017). Because the survey waves were administered at different stages and were not matched at the individual level, the quantitative component was treated as stage-specific cross-sectional evidence rather than as a longitudinal test of change. Accordingly, the analysis emphasizes distributional patterns, comparative tendencies, and carefully delimited associations rather than causal or developmental claims. The overall logic of the transfer-sensitive CIPP evaluation and the bounded LLM-assisted thematic-analysis workflow is presented in Figure 2.

This design was chosen because the project's practical purpose was not merely to describe a course but to evaluate it in a way that could support subsequent iteration. For that reason, the study integrates descriptive, interpretive, and decision-oriented layers. Statistical tendencies were not treated as self-explanatory; they were read together with narrative evidence, implementation constraints, and organizational conditions.

### 4.2 Analytic generalization and transferability

**Figure 3:**
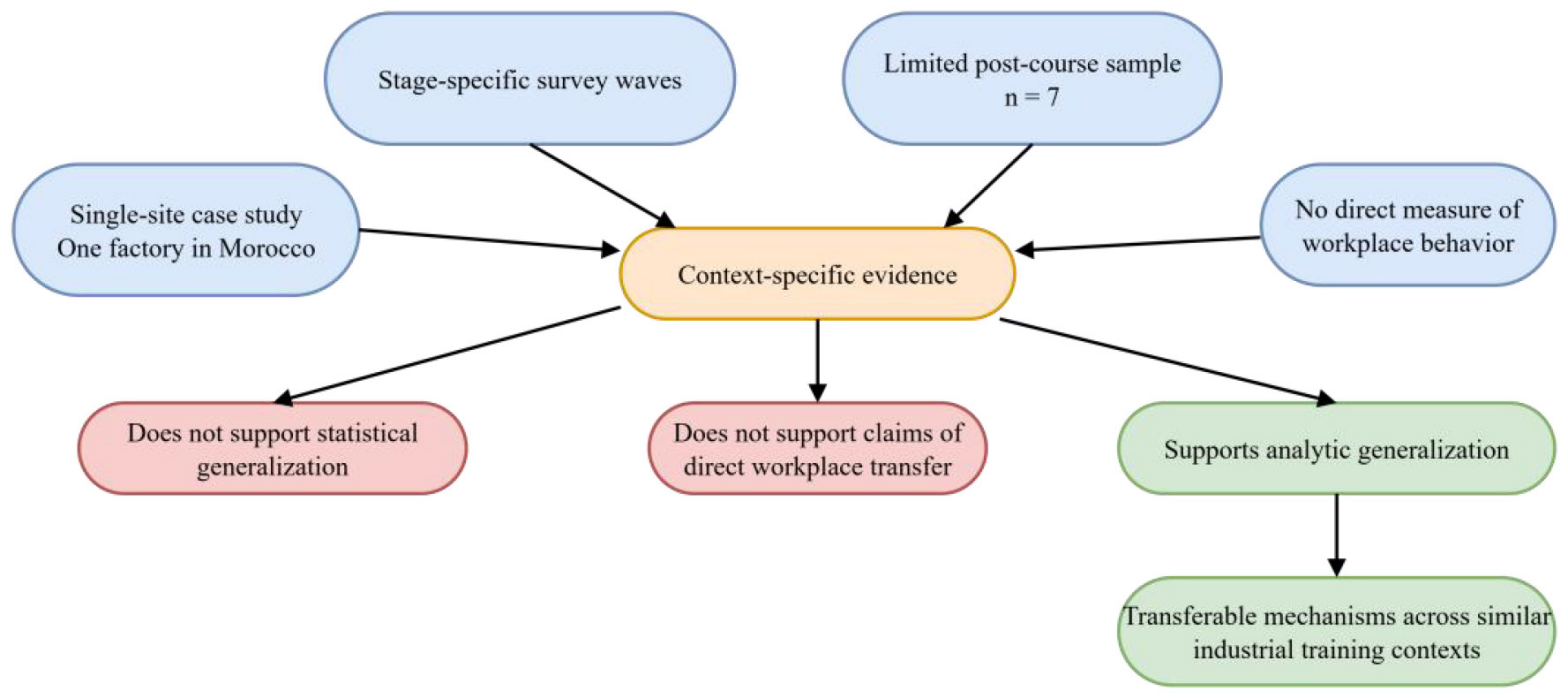
Case-study design and the interpretive scope of analytic generalization.

This study focuses on a workplace Chinese program implemented in a Chinese-managed factory in Morocco and is therefore a single-site case study. As indicated by the research design shown in Figure 3, the study is characterized by a single research setting, stage-specific survey administration, a relatively small post-course sample, and the absence of direct workplace behavior measures. For this reason, it is better suited to analyzing the key conditions and mechanisms operating within a specific training context than to serving as a basis for statistical inference across all overseas industrial training settings. Although the findings are drawn from one particular case, they still offer useful reference value for workplace language training in similar industrial contexts. In particular, they help illuminate how production pressure, learning tasks, and organizational support jointly shape training outcomes, and they can provide a basis for training design, implementation, and evaluation.

### 4.3 Data sources and measurement dimensions

**Figure 4:**
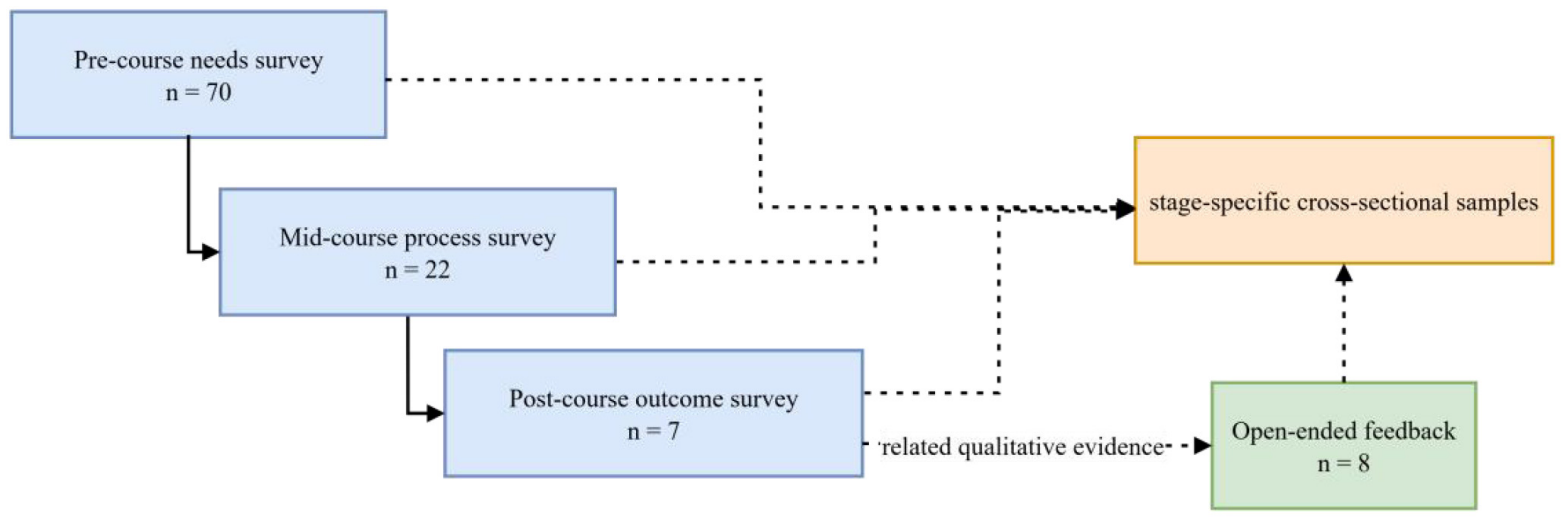
Sample sizes across different stages of data collection.

This study drew on five main sources of data. The pre-course needs questionnaire (*n* = 70) examined workplace communication scenarios, types of vocabulary needs, skill priorities, resource preferences, cultural-content needs, and preferences regarding classroom format. The mid-course process questionnaire (*n* = 22) included demographic information, learning experience, and evaluations of teaching materials, pacing, classroom climate, self-efficacy, and learning formats. The post-course outcome questionnaire (*n* = 7; open-ended feedback *n* = 8) addressed expectation fulfillment, perceived difficulty, willingness to participate, preferences for teaching modes, support needs, and suggestions for improvement, with some items having only five valid responses. These questionnaires were collected at different stages of the course and were not strictly matched at the individual level; accordingly, each wave was treated as a stage-specific cross-sectional sample rather than as a longitudinal cohort tracking the same learners over time. As shown in Figure 4, the sample sizes varied substantially across stages. The marked reduction in the post-course sample was associated with factors such as shift schedules, production peaks, personnel mobility, and the voluntary nature of questionnaire completion.

In addition, this study also collected two types of qualitative materials: a teacher interview and supervisory feedback. The teacher interview consisted of one semi-structured interview focusing on classroom implementation, learning difficulties, organizational constraints, effective activities, and directions for course revision. The supervisory feedback comprised three brief inputs from the production, quality, and human resources departments, respectively, and was used mainly to cross-check organizational conditions related to training implementation. These materials were used primarily to help interpret the patterns reflected in the questionnaire and performance data rather than as stand-alone evidence of learning effectiveness. Attendance and performance records were used to reflect learning output and participation continuity.

These measurement dimensions were aligned with the CIPP framework. Context mainly reflected learner needs related to tasks, terminology, and communication scenarios. Input mainly involved task arrangement, learning resources, and teaching materials. Process mainly reflected classroom experience, peer interaction, and organizational conditions affecting participation. Product included both learning performance and transfer-related indicators. In this study, transfer was distinguished at two levels. The first level referred to transfer-related indicators, that is, proximal conditions and signals that may support later workplace language use, such as self-efficacy, perceived task relevance, participation continuity, and performance in the training context. The second level referred to transfer outcomes, that is, directly observable workplace language behavior, such as the use of Chinese in job interactions, task completion quality, and language-mediated coordination. The present study measured the first level but did not directly measure the second. Therefore, product-level results are more appropriately interpreted as evidence of transfer-related conditions and early indicators rather than as direct proof that workplace transfer has occurred. The integration of quantitative and qualitative evidence was summarized through a joint evidence-to-decision display.

### 4.4 Quantitative analysis strategy

For multiple-choice items, the study calculated the number of selections for each option and the proportion relative to the number of respondents. For Likert-scale items, mean values were computed and visualized. Attendance and performance data were summarized mainly through means, quartiles, and grade distributions. The “classroom performance” component in the composite score was not intended to distinguish fine-grained differences in language ability. Rather, it functioned as a low-risk, participation-oriented indicator, mainly reflecting learners' in-class participation after attendance, task completion, cooperative engagement, and willingness to speak. In scoring, greater emphasis was placed on whether learners participated actively and completed classroom tasks, rather than on strict differentiation of subtle differences in language performance.

Because grade-related data were characterized by distributional skew, small sample size, and the ordinal uncertainty inherent in grade-type measures, the study used Spearman's ρ to estimate the rank-order association between attendance and overall course performance. Since attendance accounted for 10% of the composite score, the attendance–performance relationship was reported mainly as a descriptive pattern. To examine whether this correlation was driven largely by the attendance component embedded in the grading structure, the study calculated an attendance-adjusted score using the course's established scoring rule, defined as “composite score – 10× attendance rate,” and conducted a sensitivity check on that basis.

### 4.5 Qualitative analysis and credibility control

The qualitative strand used thematic analysis (Braun and Clarke, 2006). The analytic sequence included familiarization with the data, initial coding, theme aggregation, theme review, theme naming and definition, and interpretive writing. During theme development, negative-case checking was conducted by actively searching for statements inconsistent with the dominant pattern, thereby refining the boundaries of interpretation. Data triangulation across open-ended questionnaire responses, teacher interview material, and quantitative distributions was used to strengthen interpretive robustness. Because the enterprise setting involved strong anonymity requirements, quotations were minimized and de-identified before inclusion in the manuscript. The single teacher interview was treated as contextual triangulation rather than as stand-alone proof of general mechanism.

Researcher reflexivity was also important. The study explicitly avoided inferring “effectiveness” from any single source of evidence. For example, the attendance-performance relationship does not mean that attendance itself caused higher achievement; it may reflect both the structure of the composite score and the substantive importance of sustained learning investment. Likewise, because the open-ended feedback sample was small and uneven in informational density, thematic claims were supported wherever possible by multiple data sources and limited by negative-case checking to reduce the risk of overgeneralization.

### 4.6 LLM-assisted analysis protocol

To improve consistency in the preliminary organization of open-ended responses while preserving researcher control over interpretation, this study introduced a bounded LLM-assisted workflow into the qualitative analysis (Nguyen and Welch, 2026). The model used was Anthropic's Claude Opus 4.6, accessed through the Anthropic platform, and all prompts, outputs, and processing dates were preserved in the audit trail. The model was used only for early-stage code organization and evidence classification. It was not involved in generating final themes, nor did it replace the researchers' close reading, comparison, and interpretation of the original materials.

In practical implementation, all qualitative materials were de-identified before being entered into the model. Names, employee identifiers, and enterprise-sensitive information were removed, and only the minimum text segments necessary for analysis were retained. Model outputs were limited to candidate codes, brief summaries, evidence-linked theme suggestions, and uncertainty markers. The researchers then manually reviewed, revised, merged, or deleted all suggestions and developed the working codebook on that basis. Detailed information on prompt design principles, constraint rules, and output formats is provided in **SI-5** of the Supporting Information.

To enhance methodological transparency, this study also incorporated a human–LLM comparison check at the code-organization stage. Two researchers first independently coded a subset of the open-ended materials, after which Claude Opus 4.6 generated candidate codes for the same subset under the same evidence-constrained conditions. The two sets of coding were then compared at the category level, and agreement was reported both between the researchers and between human coding and LLM-assisted coding using Cohen's kappa. All disagreements were resolved through researcher discussion, return to the original texts, and negative-case checking. Table 1 presents an example of coding before and after LLM assistance based on teacher interview excerpts, showing that the main contribution of the LLM lay in refining and regrouping code labels, while final coding decisions remained grounded in the original context and were made by the researchers.

**Table 1:** Example of coding before and after LLM assistance based on teacher interview excerpts.

Raw response excerpt	Human initial code	LLM-suggested code	Final code
“We turned the larger goal into phased smaller goals and moved step by step.”	goal–need alignment	workplace-driven target setting; phased scaffolding	needs-based goal alignment with staged scaffolding
“They now hope to start more from everyday life, because that creates less pressure.”	easier entry point	everyday-life entry; pressure reduction	preference for life-based entry to reduce learning pressure
“If learners miss classes… they lose confidence, and then they may not want to come.”	absence affects continuity	lagging behind → confidence loss → withdrawal risk	absence-related discontinuity and confidence erosion

The study maintained a cautious stance toward LLM assistance throughout. Model outputs may introduce anchoring bias or structuring bias by compressing heterogeneous responses into overly tidy categories. To reduce this risk, prompt design and output scope were tightly constrained, all suggestions were manually adjudicated by the researchers, and a complete audit trail was preserved. Accordingly, the role of the LLM in this study was limited to methodological support; its outputs were treated neither as neutral nor as self-validating conclusions.

### 4.7 Ethics and data governance

Evaluation of language training in enterprise settings involves both organizationally sensitive information and individual privacy. To reduce risk, the study followed a minimum-necessary-data principle. Only de-identified data directly relevant to the research questions, including questionnaire text, interview excerpts, and statistical aggregates, were used for analysis. The manuscript does not include personally traceable names, employee numbers, or department identifiers, and performance and attendance data were anonymized during export and sharing.

The LLM-assisted component was governed by the same principle. Input text was limited to minimal necessary excerpts, and prompts explicitly instructed the model not to fabricate or complete information that did not appear in the data. An audit trail was preserved to support methodological transparency and subsequent review. In this way, the study treated data governance as integral to research design rather than as an afterthought.

## 5 Results

### 5.1 Context: needs structured by task, terminology, and scenario

**Figure 5:**
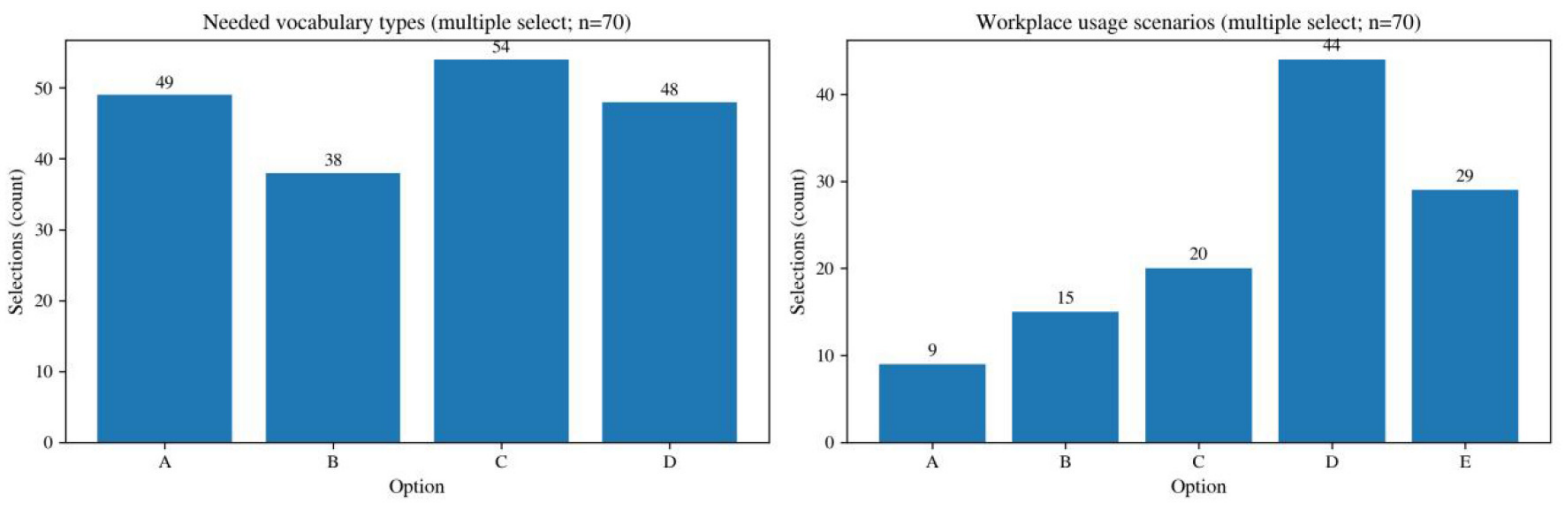
Pre-course needs: vocabulary types and usage scenarios (multiple response, *n* = 70).

The pre-course needs survey indicates that learners' expectations for workplace Chinese training were strongly concentrated on language resources that could be used directly at work (Figure 5). In terms of vocabulary needs, office-related daily vocabulary ranked first (54 selections, 77.1%), followed by industry-specific terminology (49 selections, 70.0%) and etiquette/social vocabulary (48 selections, 68.6%). This pattern suggests that learners were not seeking only “sentences that can be used on site,” but also terminology domains robust enough to support professional interaction. With respect to scenarios, “daily communication with Chinese colleagues or clients” had the highest selection rate (44 selections, 62.9%), while 29 respondents (41.4%) selected “all of the above,” indicating that learners saw Chinese as a cross-task collaboration tool rather than as a narrowly specialized add-on.

In terms of skill priorities, speaking and listening occupied the core of learner demand, accompanied by a substantial desire for “overall improvement.” This structure implies that the entry phase of the program should focus on building a communicative base that enables learners to speak and understand, before gradually introducing more formal written genres. Learning-preference data further support this interpretation. Preference for multimedia-assisted vocabulary learning reached 37.1%, and scenario simulation was rated “very necessary” by 25.7% of respondents. Regarding cultural content, 71.4% reported that they “very much hoped” for the inclusion of traditional culture. Culture can therefore serve as a motivational and interactive resource, but it should not be detached from work tasks. A more functional approach is to embed “micro-units” of culture into collaborative events, such as the pragmatic background of polite expression or the social use of festival-related topics.

These findings suggest that learner needs are best interpreted not as isolated requests for lexical content, but as an integrated structure of task, terminology, and scenario. Learners expected language support that would help them complete workplace tasks, handle recurrent interactional situations, and participate more confidently in cross-cultural collaboration. This pattern provides a strong rationale for organizing course content around collaborative events rather than around decontextualized vocabulary lists alone. These needs patterns and their corresponding teaching implications are summarized in Table 2.

**Table 2:** Needs analysis and teaching implications (*n* = 70).

Dimension	Key findings (%)	Teaching implication
Vocabulary domains	Office daily vocabulary 77.1%; industry terminology 70.0%; etiquette vocabulary 68.6%	Use high-frequency collaborative expressions as the main spine, then layer in terminology by job domain
Scenarios	Daily communication 62.9%; all scenarios 41.4%	Organize tasks around collaborative events: asking, clarifying, confirming, and reporting
Vocabulary-learning mode	Multimedia 37.1%; contextual sentences 57.1%; gamification 54.3%	Build a vocabulary scaffold combining audio, contextual sentences, and mini-games
Scenario simulation	Very necessary 25.7%	Allocate a high proportion of class time to role-play and scenario dialogue practice
Cultural content	Very much hope to include 71.4%	Integrate cultural micro-units into tasks to support interaction and engagement

### 5.2 Input: course usability and the tension of pacing

**Figure 6:**
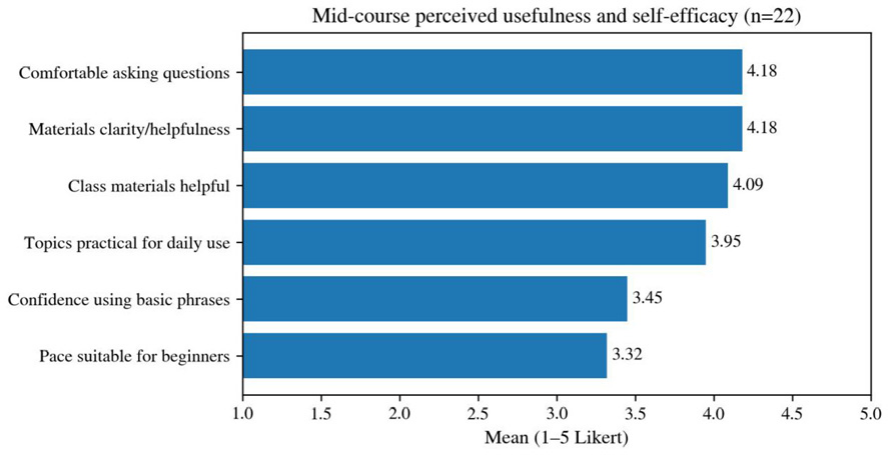
Mean mid-course ratings of course usability and self-efficacy (Likert 1–5, *n* = 22).

The mid-course questionnaire (*n* = 22) indicates generally positive evaluations of the course's usability. As shown in Figure 6, learners rated material clarity, usefulness, and classroom comfort highly, whereas pacing received the lowest mean rating. Specifically, the mean scores were 4.18 for material clarity, 4.18 for usefulness, 4.09 for overall material support, and 4.18 for comfort in asking questions. These results suggest that both the instructional resources and the classroom climate helped lower the speaking threshold for adult beginner learners. At the same time, “pacing appropriate for beginners” received the lowest mean score among all Likert-scale items (3.32). This pattern points to a clear tension between the pace of course progression and the heterogeneous needs of a beginner cohort, particularly when work-related absences disrupted continuity of participation and slowed cumulative learning.

From the perspective of input design, this finding suggests that course investment should not be understood solely in terms of textbook quality, lesson planning, or task content. Recovery mechanisms are also part of input. A course must answer not only what to teach, but how a learner can return to the learning track after absence. If such mechanisms are missing, even a high-quality course may gradually lose some learners as gaps in pacing accumulate and classroom participation becomes more difficult. In that sense, recoverability is a design resource rather than merely a logistical afterthought.

The result also clarifies a common dilemma in enterprise language education. Programs often seek rapid, visible progress because employers and learners alike value immediate utility. Yet acceleration can widen differences among learners, particularly in contexts with irregular attendance. The design challenge is therefore not simply to slow down or speed up, but to create pacing that can absorb disruption without collapsing coherence.

### 5.3 Process: support mechanisms and obstacles to learning persistence

The post-course questionnaire and open-ended feedback reveal the main mechanisms shaping sustained participation. On the supportive side, learners expressed strong approval of classroom formats that actively stimulated engagement. In the post-course survey, 71.4% of respondents reported that the course had “strongly stimulated participation,” while group collaboration and gamified learning were jointly identified as the most preferred classroom modes (both 71.4%). This suggests that low-risk opportunities for language output and peer interaction can reduce the psychological cost of speaking while increasing learners' emotional investment. Such activities may also make progress more visible, thereby strengthening the perceived value of continued participation.

**Figure 7:**
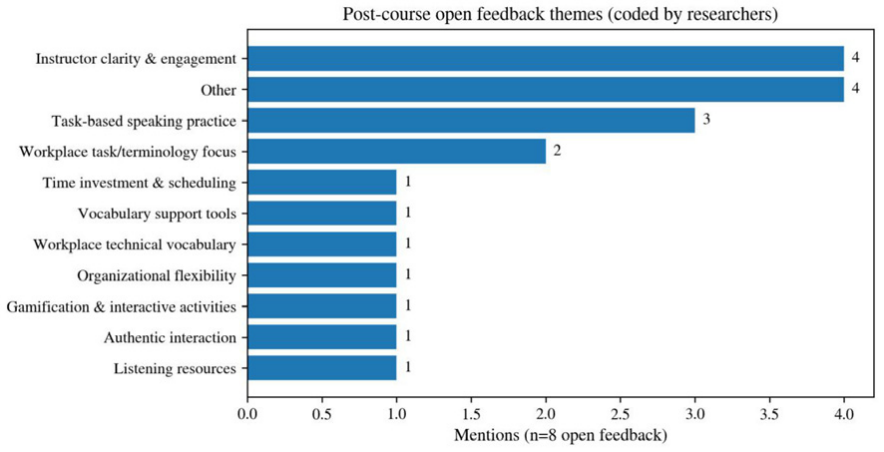
Distribution of themes in post-course open-ended feedback (researcher coding, *n* = 8).

From the thematic distribution of the open-ended feedback, the most frequently mentioned themes included instructor clarity and engagement, task-based speaking practice, and a teaching focus on workplace tasks and terminology (see Figure 7). This indicates that learners' positive evaluations of the course were shaped not only by the classroom atmosphere itself, but also by the clarity of instructional delivery and the degree to which task-oriented content aligned with actual workplace scenarios. At the same time, themes such as listening resources, opportunities for authentic interaction, time scheduling, organizational flexibility, and vocabulary support tools were mentioned less frequently, yet they still reflect learners' practical need for out-of-class support and compensatory mechanisms in sustaining participation.

Support needs outside class were concentrated around lightweight follow-up resources and social support structures. In the item asking what kinds of learning support were most needed (*n* = 7), vocabulary lists, cultural videos, and practice software each received 42.9% of responses. In another item with five valid responses, “learning community” accounted for 60% and “after-class materials” for 40%. Although the sample size was small, this pattern still suggests that learners wanted support structures beyond formal classroom hours that could help maintain continuity in learning. The teacher interview echoed this pattern: on the one hand, production schedules and missed classes were identified as the main obstacles to continuity; on the other hand, cultural activities and interactive tasks were viewed as important sources of sustained engagement.

Taken together, these findings suggest that learning persistence was not determined by motivation alone. Rather, it emerged from the interaction among engaging in-class design, manageable out-of-class support resources, and the availability of effective support mechanisms after disruption. In a work-constrained learning ecology, whether learners can sustain participation depends on whether they are able to reconnect with the course repeatedly in ways that feel both feasible and worthwhile.

### 5.4 Attendance and performance: descriptive pattern and sensitivity check

**Figure 8:**
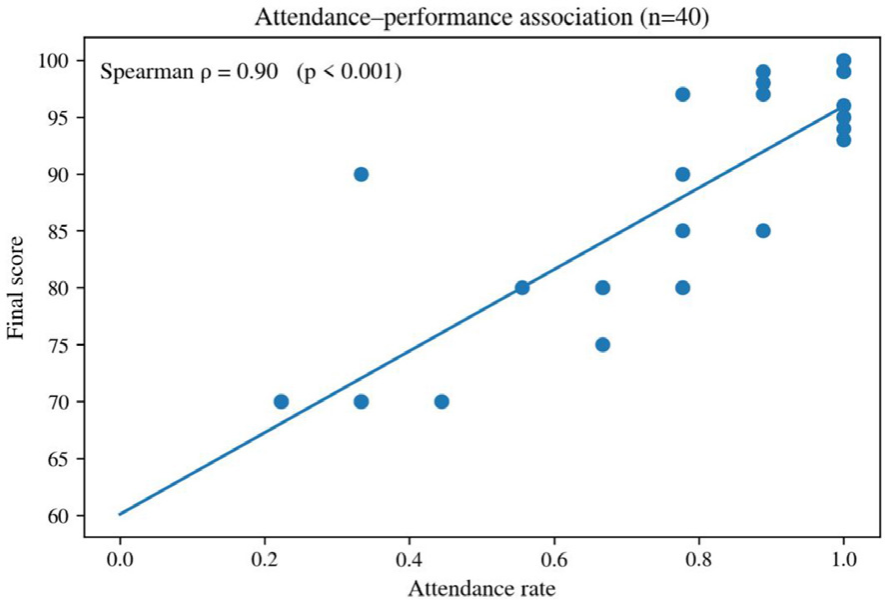
Association between attendance rate and composite score (descriptive pattern, *n* = 40).

Across all 52 registered participants, mean attendance was 0.523 and the median was 0.444, indicating marked dispersion. Approximately 27.5% had attendance rates of 0.80 or above, while 35.3% had attendance rates of 0.30 or below. Among the 40 learners included in the valid performance analysis, attendance and composite score were strongly positively associated (Spearman's ρ = 0.90, *p* < 0.001; Figure 8). Because attendance contributed 10% of the composite score, this raw association contains a mechanical component. A sensitivity check using an attendance-adjusted score (composite score – 10× attendance rate) still produced a substantial positive association (ρ = 0.78, *p* < 0.001). The result should therefore be read as evidence of covariation between participation continuity and achievement-related performance, not as a causal estimate.

The dispersion in attendance is itself important. It means that the same course delivered very different effective learning doses to different learners. Under such conditions, the average effect of any instructional design will be diluted at the group level, even if the design is pedagogically sound. This helps explain why positive evaluations of materials or classroom atmosphere do not automatically translate into uniformly strong results. In enterprise settings, continuity of participation is not merely a background variable; it is part of the mechanism by which learning opportunity is differentially realized.

For evaluation, the implication is clear. Programs seeking stronger overall outcomes must treat attendance not only as a record of presence but as an indicator of whether the learning ecology is workable for participants. Where attendance is highly dispersed, governance and course design must jointly address effective dose rather than assuming that all enrolled learners have received comparable exposure.

### 5.5 Qualitative themes: from wanting to learn to being able to use

Synthesizing the open-ended feedback and interview data, the study identified three core themes. First, task-based output functioned as the key hinge for building usable language. Learners repeatedly emphasized scenario dialogue, speaking practice, technical content, and work-related Chinese, indicating that they wanted executable scripts and repeated rehearsal directly tied to work events. Second, organizational time constraints constituted a structural obstacle to persistence. Absence led to broken pacing, which in turn weakened self-efficacy and willingness to continue; where compensation mechanisms were absent, participation gaps widened and reduced participation became more likely. Third, community and resources formed the bridge between classroom learning and workplace use. Learners asked for after-class materials, peer-support space, and opportunities to interact with native speakers, all of which can create lower-risk settings for trial use.

These themes are analytically useful because they connect motivation, design, and governance. Learners did not merely want “more content.” They wanted opportunities to rehearse, structures that would help them recover from interruption, and social or material scaffolds that would make workplace application feel possible. Negative-case checking also suggested an important boundary: not all learners explicitly articulated transfer to real workplace use, and not all feedback was equally information-rich. For that reason, the themes should be read as interpretive patterns supported by triangulation rather than as claims of universal experience. The themes, evidence sources, and corresponding improvement directions are summarized in Table 3.

**Table 3:** Themes, evidence sources, and improvement directions.

Theme	Evidence source	Illustrative quotation	Direction for improvement
Need for task-based speaking practice	Open-ended feedback	“Make dialogues and practice speaking more.”	Increase scenario scripts, role-play, and repeated rehearsal; embed terminology in tasks
Insufficient listening and input resources	Open-ended feedback	“Add more listening audios.”	Build a leveled audio library and pair it with micro-tasks (shadowing/retelling)
Need for authentic interaction opportunities	Open-ended feedback	“Arrange activities with native speakers.”	Introduce language-partner mechanisms and weekly workplace task challenges
Time and policy constraints	Interview + feedback	“Flexibility regarding absences...” and teacher comments on missed classes	Build unit learning packs and re-entry tasks; consider more flexible absence rules and replay resources
Classroom climate and clarity of teaching	Feedback + interview	“Very clear and good/Engaging.”	Maintain structured explanation with low-pressure interaction; strengthen feedback and encouragement

### 5.6 Product: outcomes and transfer-related indicators

The post-course outcome survey suggests that the course generally met or exceeded learners' expectations. Specifically, 57.1% reported that the course had “far exceeded” expectations, while the remaining 42.9% indicated that it had “met” expectations. Most respondents also considered the difficulty level appropriate (57.1%) or somewhat challenging in a manageable way (28.6%). Because these post-course responses were drawn from a very small stage-specific sample (*n* = 7, with some items based on *n* = 5), they should be interpreted as exploratory signals rather than as stable population estimates. Even so, the response distributions indicate that the program was experienced as neither trivial nor overwhelmingly difficult, a balance that is especially important for sustaining adult learner participation. These post-course distributions, including perceived outcomes, classroom preferences, and support needs, are summarized in Table 4.

**Table 4:** Post-course distributions.

Indicator	*N*	Distribution of responses (%)
I.1 Fulfillment of expectations	7	Far exceeded 57.1%; Met 42.9%; Partly met 0.0%; Did not meet 0.0%
I.2 Match of difficulty	7	Too easy 14.3%; Appropriate 57.1%; Somewhat difficult 28.6%; Too difficult 0.0%
II.6 Stimulation of participation	7	Strongly stimulated 71.4%; Stimulated 28.6%; Neutral 0.0%; Not stimulated 0.0%
II.8 Preferred classroom format (multiple response)	7	Group collaboration 71.4%; Scenario simulation 28.6%; Gamified learning 71.4%; Traditional lecture 14.3%
III.10 Most needed learning support (multiple response)	7	Vocabulary list 42.9%; Cultural videos 42.9%; Practice software 42.9%; Supplementary reading 28.6%

**Figure 9:**
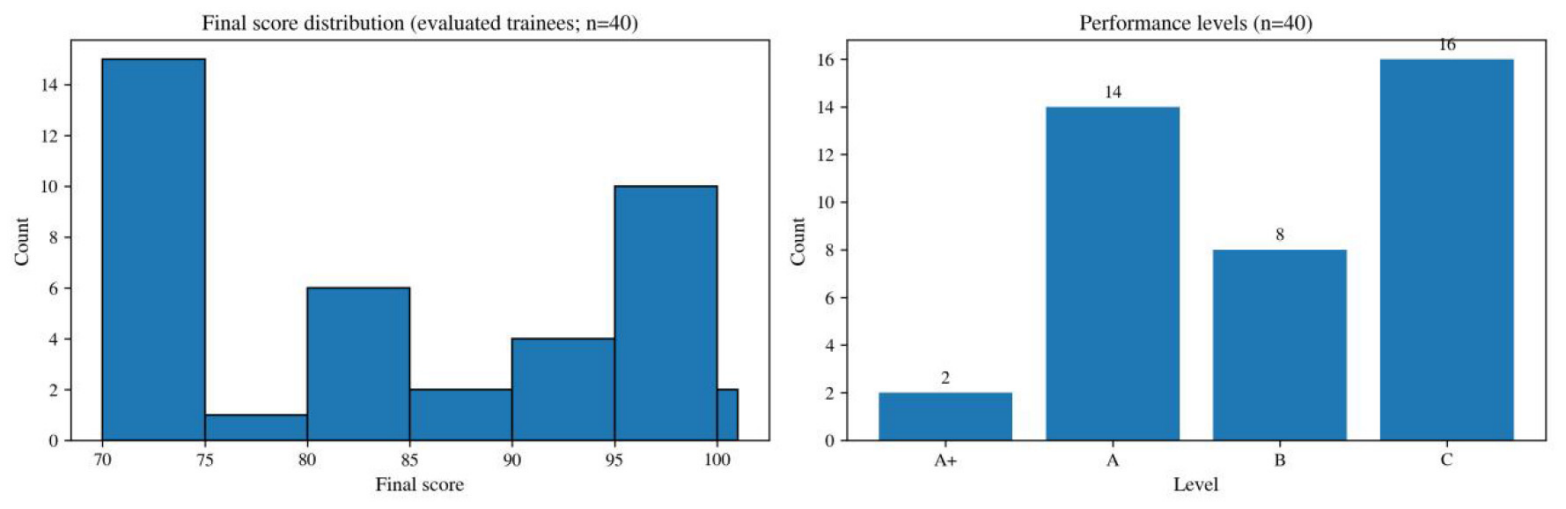
Distribution of overall course scores and grade composition (40 valid score records).

In terms of objective performance, learners with valid evaluation records had a mean composite score of 82.9 (SD = 12.0), with a median of 80 and a score range of 70–100. The grade distribution was A+ = 2, A = 14, B = 8, and C = 16 (see Figure 9). Overall, the introductory course generated a certain level of learning output, although a considerable number of learners still remained at the C level. Scores on the classroom-performance component were mostly concentrated in the higher range and therefore had limited discriminating power. This component was designed mainly to encourage participation, reduce speaking anxiety, and strengthen task engagement; accordingly, scoring placed greater emphasis on classroom participation and task completion than on fine-grained differentiation in language performance. In an adult beginner context, such an indicator is more likely to produce a clustering of high scores. As a result, differences in the composite score were driven mainly by test performance and attendance, while the classroom-performance component is better understood as a supportive indicator of participation rather than as a highly discriminating measure of language ability.

With respect to transfer, the present study deliberately interprets product evidence as transfer-related indicators rather than as definitive proof of workplace behavior change. The relevant signals include self-reported increases in confidence, positive evaluations of task-oriented learning, sustained participation among some learners, and measurable performance output. These indicators do not document workplace transfer itself. Instead, they suggest that the course created conditions under which later workplace use may become more likely if supported by continued practice opportunities and organizational protection for learning.

### 5.7 Triangulation from teacher and supervisory perspectives

The teacher interview and supervisory feedback provided additional perspectives for interpreting the quantitative findings and open-ended responses. The teacher noted that class hours were limited and that heavy work demands substantially reduced time for in-class practice. The production supervisor likewise pointed out that the main challenge was not a lack of willingness to learn, but the fact that shift schedules and production tasks repeatedly interrupted learning continuity. This suggests that, in production-constrained enterprise settings, whether training can be sustained depends not only on classroom arrangements, but also on the tension between work rhythms and learning rhythms.

Second, regarding effective activities, the teacher considered cultural activities, interactive tasks, and a relatively relaxed classroom atmosphere helpful for increasing learner participation, which is broadly consistent with the post-course survey findings showing learners' preference for gamified learning and group collaboration. The quality supervisor further stressed that the most valuable training should directly serve workplace communication, such as confirming information, reporting problems, and requesting help. This indicates that course effectiveness depends not only on a positive classroom experience, but also on the degree to which training content aligns with real workplace tasks.

Third, regarding improvement pathways, the teacher noted that shift patterns and personnel mobility weakened learning continuity, and therefore emphasized the need for catch-up materials, re-entry arrangements, and cross-group support to improve recoverability. From an organizational-support perspective, the human resources supervisor also pointed out that stable training effects depend not only on what is taught in class, but also on whether employees can continue participating under work pressure and whether protected time, catch-up resources, and supervisor support are available. Overall, the teacher and supervisory feedback is not used to prove that stable workplace transfer has already occurred. Rather, it more clearly indicates that organizational support, opportunities for practice, and recoverable learning design are key conditions shaping whether training can move from “wanting to learn” to “being able to use.” Relevant feedback is summarized in Table 5.

**Table 5:** Summary of supervisory feedback.

Role	Feedback
Teacher	The biggest difficulty at present is still the limited class hours combined with heavy work demands, so practice is not sufficient. Cultural activities, interactive tasks, and a more relaxed classroom atmosphere can encourage participation, but shift schedules and personnel mobility easily interrupt learning continuity. Going forward, progress will need to be reconnected through catch-up materials, return-to-class arrangements, and cross-group support.
Production supervisor	This course is helpful for frontline communication, especially in understanding simple instructions, confirming tasks, and reducing hesitation in communication. The main problem is not a lack of willingness to learn, but the fact that shift schedules and production arrangements easily disrupt learning continuity.
Quality supervisor	What learners need most are expressions that can be used directly for workplace communication, such as confirming information, reporting problems, and asking for help. Classroom practice is useful to some extent, but for it to truly transfer into work performance, more job-relevant practice opportunities are still needed.
Human resources supervisor	The key issue is not only what the course teaches, but whether employees can continue to participate under work pressure. If time protection, catch-up resources, and supervisory support can be further strengthened, the training effect will be more stable.

## 6 Discussion

The needs analysis showed that learners in the factory setting were primarily concerned with expressions, terminology, and interactional routines that could be directly used in concrete work tasks and real communicative situations. This suggests that the effectiveness of workplace Chinese training cannot be evaluated solely in terms of classroom outcomes, but also depends on whether the instructional content is aligned with actual work demands, whether participation can be sustained, and whether the conditions for later transfer are in place. In terms of course design, instruction should first be organized around reusable interactional frames, such as asking, clarifying, confirming, reporting, and requesting support, and only then gradually extended to more specialized terminology associated with particular roles or departments (Ellis, 2003). These findings indicate that needs analysis is not only the starting point for course design, but can also serve as an important basis for identifying course problems and adjusting instructional priorities.

**Figure 10:**
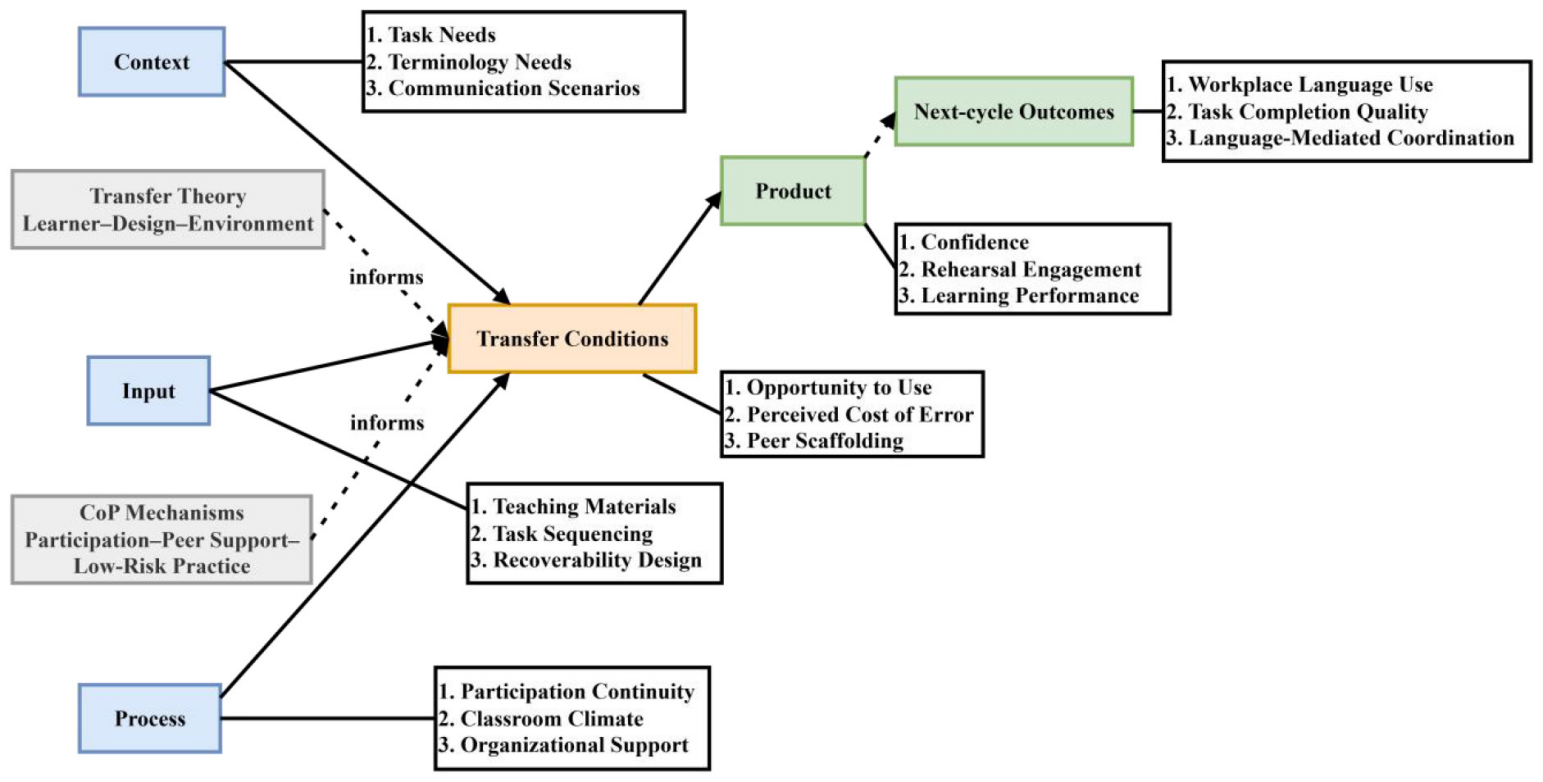
Integrated framework of CIPP, transfer conditions, and CoP.

Figure 10 places CIPP, transfer conditions, and CoP mechanisms within the same analytical structure in order to clarify the relationships among the key issues identified in this study. In this case, the most important issue was not the level of learner satisfaction, but the combined effect of pacing difficulty and irregular attendance. As shown in the figure, Context, Input, and Process are not separate layers; rather, they jointly shape whether the conditions for later workplace language use are present, including whether learners have opportunities to use Chinese, whether they can tolerate the pressure associated with making mistakes, and whether peer support is available. Both the attendance–performance correlation and the sensitivity check after removing the attendance component from the composite score suggest that participation continuity is not merely a formal variable, but one that has substantive implications for learning. In workplace language training under strong production constraints, absence is not simply a matter of classroom management; it interrupts cumulative learning, weakens learner confidence, and affects subsequent participation. For this reason, the recoverability of catch-up and re-entry mechanisms becomes a key link connecting Input, Process, and Product.

Within this framework, the Product-level findings reflect transfer-related indicators rather than workplace transfer outcomes themselves. What the present study identifies are mainly proximal signals that may support later transfer, including learner confidence, willingness to engage in task-based rehearsal, measurable learning performance, and sustained demand for more authentic interaction opportunities. More stable workplace language use, by contrast, still remains to be examined in future research. The current evidence is not sufficient to conclude that stable workplace language use has already been established. The teacher interview and open-ended responses both suggest that, before classroom performance can develop into routine language use in everyday work, learners still need more opportunities for practice, lower-risk environments for trial use, and stronger support after absence. On this basis, Table 6 highlights several priorities for course revision, including organizing teaching around collaborative events, embedding terminology by role or process, introducing catch-up and re-entry mechanisms, and expanding structured interaction opportunities such as role-play, group tasks, peer support, and micro-task rehearsal. In industrial settings, the core challenge of workplace Chinese training lies not only in linguistic difficulty itself, but also in how to maintain continuity of learning under conditions of frequent disruption and gradually create the conditions for later transfer.

**Table 6:** Joint display of evidence, interpretation, and course-revision priorities.

Evidence pattern	Interpretation	Course-revision priority	Example practice
High frequency of scenarios and collaborative events	Need-task alignment is central to perceived relevance	Use collaborative events as the instructional backbone	Reusable asking-clarifying-confirming-reporting sequences with politeness routines
Strong terminology demand	General communication and domain terminology need to develop together	Layer terminology by role or process	Contextualized vocabulary lists and short sentence frames by department
Pace differentiation and absence	Participation continuity is a design variable, not only an administrative issue	Build recoverability into the course	Unit packs, catch-up tasks, replay resources, and peer buddies
Preference for interaction and community support	Low-risk rehearsal and peer support reduce the cost of speaking	Expand structured interaction opportunities	Role-play, group tasks, language partners, and micro-tasks
Limited direct transfer evidence	Product indicators should be interpreted cautiously	Add follow-up measures closer to workplace use	Anonymous workplace micro-tasks, delayed follow-up checks, and peer or supervisor feedback

From a methodological perspective, the main value of this study lies in using CIPP as an analytical framework to examine learning needs, course design, participation conditions, and Product-level indicators within a single structure. In doing so, it brings originally scattered process and outcome evidence into a unified explanatory chain and further links that chain to course-revision priorities. The role of the LLM in this study was limited to methodological support, mainly for the preliminary organization of short qualitative texts. Its use was grounded in de-identification, constrained prompting, researcher adjudication, and the preservation of an audit trail, and interpretive authority remained with the researchers throughout. Although the study adopted a single-site case design, implemented questionnaires in stage-specific rather than individually matched waves, and did not directly measure workplace behavior, these features do not diminish its value as a context-sensitive analysis. Rather, they allow the study to focus more directly on the conditions and mechanisms that matter most in the actual implementation of training, especially task relevance, pacing, participation continuity, peer support, and organizational time protection. For workplace language training in similar industrial settings, these factors have both explanatory significance and practical value for course design, implementation, and evaluation. Future research may build on this by adopting longer-term tracking designs and incorporating evidence closer to real workplace use, such as supervisory feedback, workplace observation, and task logs, in order to further examine how these mechanisms develop into relatively stable language transfer outcomes under different organizational conditions.

## 7 Conclusion

This study examined workplace Chinese training in a Chinese-managed factory in Morocco through a single-site mixed-methods case analysis based on a transfer-sensitive CIPP evaluation framework. The findings show that learner needs were mainly organized around concrete work tasks and real interactional situations, that the continuity of participation was clearly affected by instructional pacing and absence, and that the course outcomes were reflected primarily in proximal indicators related to transfer rather than in direct evidence that stable workplace language transfer had already been established. Under conditions of production constraint, the effectiveness of workplace Chinese training should therefore not be judged solely by satisfaction or test scores, but also understood in relation to task relevance, participation continuity, learning support mechanisms, and organizational arrangements.

The study further identified the key conditions and mechanisms that shape whether workplace language training can function effectively in a real industrial setting. Task relevance, instructional pacing, participation continuity, peer support, and organizational time protection all emerged as important factors influencing whether training can gradually develop into actual use. Teacher interviews and supervisory feedback further suggest that organizational support, practice opportunities, and recoverable learning design are crucial conditions for moving training from classroom learning toward actual use. The CIPP framework provided a unified analytical structure for bringing together otherwise fragmented evidence on needs, process, and outcomes, and also offered a basis for identifying priorities for course revision. Future research may build on this by combining longer-term tracking designs with evidence that is closer to real workplace use, such as supervisory feedback, workplace observation, and task logs, in order to further examine how these mechanisms develop into relatively stable transfer outcomes under different organizational conditions.

## Data Availability

The raw data supporting the conclusions of this article will be made available by the authors, without undue reservation.
