# Supplementary material for: Workplace Chinese training in a Chinese-managed factory in Morocco: a transfer-sensitive CIPP evaluation
Source: Front Psychol. 2026 May 14;17:1835254. doi: 10.3389/fpsyg.2026.1835254 (PMC13217399; doi:10.3389/fpsyg.2026.1835254)
Supplement: Supplementary file 1 [file Table_1.docx]

Supplementary Material

# SI Overview

This Supporting Information provides additional methodological detail and worked examples referenced in the main manuscript, including (i) an expanded description of data sources and measurement dimensions; (ii) the composite-score computation and attendance-adjusted sensitivity check; (iii) an excerpted qualitative codebook and triangulation notes; and (iv) prompt templates and audit-trail fields for the bounded LLM-assisted workflow.

# SI-1. Data Sources and Measurement Dimensions

Tables S1a–S1b expand Section 4.2 by summarizing each data source, its stage, analytic purpose, and examples of measured constructs as described in the manuscript. Survey waves are stage-specific snapshots rather than matched observations.

Table S1a. Survey sources and measurement dimensions

| Data source | Stage / timing | n | Primary constructs captured | Examples (from manuscript) |
| --- | --- | --- | --- | --- |
| Pre-course needs questionnaire | Pre-course | 70 | Scenarios; vocabulary domains; skill priorities; resource & culture preferences; format expectations | Top vocab: office daily; industry terms; etiquette/social. Scenarios: daily comms; multi-scenario. |
| Mid-course process questionnaire | Mid-course | 22 | Demographics; learning history; ratings (materials, pacing, climate, self-efficacy); format preferences | Likert (1–5) means; pacing item; comfort asking questions; self-efficacy. |
| Post-course outcome questionnaire + open-ended feedback | Post-course | 7 (+ open-ended n=8; some items n=5) | Expectation fit; difficulty match; participation stimulation; preferred modes; support needs; suggestions | Preferred: group collaboration, gamified learning. Needs: vocab lists, videos, apps; more listening, native-speaker interaction. |

Table S1b. Non-survey sources and key measurement elements

| Non-survey source | n | Key information captured (from manuscript) |
| --- | --- | --- |
| Teacher semi-structured interview (contextual triangulation) | 1 | Constraints: reduced contact hours, work pressure, shift changes, mobility. Emphasis on repeated practice, relaxed climate, and more use opportunities. |
| Attendance and performance records | 40 valid score records (52 registered) | Weights: attendance 10%, classroom 25%, assignments 10%, tests/exams 55%; classroom-performance near-uniform full marks. |

# SI-2. Composite Score Construction and Attendance-Adjusted Sensitivity Check

The program’s published weighting rule combined four components into a composite score: attendance (10%), classroom performance (25%), assignments (10%), and tests/examinations (55%). Classroom performance in the analyzed cycle was reported as almost uniformly full marks, so performance differences were mainly driven by tests and attendance.

Let A be attendance rate (0–1), CP be classroom-performance proportion (0–1), HW be assignment proportion (0–1), and T be test proportion (0–1).

Composite score = 10·A + 25·CP + 10·HW + 55·T.

Because attendance contributes directly to the composite score, the correlation between attendance and composite performance contains a mechanical component. The sensitivity check removes this embedded component using: Attendance-adjusted score = Composite score − 10·A.

# SI-3. Quantitative Analysis Notes

Multiple-choice (including multiple-response) items were summarized by counts and proportions relative to the number of respondents. Likert-scale items were summarized using mean values and plotted for interpretability. Attendance and performance distributions were summarized via means, quartiles, and grade frequencies. Because of small samples, skewed distributions, and ordinal uncertainty in grade-like outcomes, Spearman’s rho (ρ) was used to describe rank-order association between attendance and course performance, supplemented by the attendance-adjusted sensitivity check described in SI-2.

# SI-4. Qualitative Codebook Excerpt and Triangulation Notes

Thematic analysis followed the familiarization → initial coding → theme aggregation → review → naming/definition → interpretive writing sequence. Negative-case checking was used to refine theme boundaries by actively searching for statements that diverged from the dominant pattern. Triangulation relied on convergence across open-ended feedback, teacher interview context, and quantitative distributions.

Table S2. Codebook excerpt with operational definitions and examples

| Theme (reported) | Working subcodes | Operational definition | Example excerpt (de-identified) | Linked improvement direction |
| --- | --- | --- | --- | --- |
| Task-based output as hinge for usable language | Scenario dialogue preference; speaking practice; executable scripts; terminology-in-task | Learners prioritize repeated, low-risk rehearsal of workplace-relevant dialogues and task scripts over decontextualized coverage. | “Make dialogues and practice speaking more.” | Increase scenario scripts, role-play, and repeated rehearsal; embed terminology in tasks. |
| Time constraints and absence-related discontinuity | Workload pressure; shift changes; broken pacing; recoverability after absence | Production rhythm and irregular attendance disrupt learning continuity; learners need re-entry mechanisms to prevent pacing gaps from accumulating. | “Flexibility regarding absences...” (plus teacher emphasis on missed classes). | Build unit learning packs and re-entry tasks; strengthen replay resources and peer-buddy support. |
| Community and resources as bridge to later workplace use | After-class materials; lightweight practice tools; peer support space; native-speaker interaction opportunities | Learners request continuity structures beyond class hours, including materials and social support that reduce speaking cost and enable trial use. | “Add more listening audios.” / “Arrange activities with native speakers.” | Build leveled audio library with micro-tasks; introduce language-partner mechanisms and workplace micro-challenges. |

# SI-5. Bounded LLM-Assisted Workflow: Prompt Templates, Output Schema, and Audit Trail

## SI-5.1 De-identification and minimum-necessary input rule

Before any LLM-assisted step, text was de-identified by removing names, employee numbers, and enterprise-sensitive information, and by retaining only minimally necessary excerpts. This governance rule was applied consistently across all LLM runs.

## SI-5.2 Prompt Template A: Candidate coding for short open-ended responses

SYSTEM: You are assisting with preliminary organization of de-identified short qualitative responses. Do not fabricate content.
USER:
You will receive a list of de-identified response excerpts. For each excerpt:
1) Propose 1–3 candidate codes (short phrases).
2) Provide an evidence identifier (use the provided ID).
3) Add an uncertainty marker: LOW / MEDIUM / HIGH.
Output must be valid JSON and contain only the fields specified.

INPUT FORMAT:
[{ "evidence_id": "<ID>", "excerpt": "<TEXT>" }, ...]

OUTPUT JSON SCHEMA:
{ "items": [ { "evidence_id": "<ID>", "candidate_codes": ["..."], "uncertainty": "LOW|MEDIUM|HIGH", "notes": "<brief>" } ] }

## SI-5.3 Prompt Template B: Theme aggregation with boundary conditions

SYSTEM: You are assisting with grouping researcher-approved codes into candidate themes. Do not invent themes not supported by codes.
USER:
Given a list of approved codes and short de-identified supporting excerpts, group codes into 3–6 candidate themes.
For each theme, provide:
- Theme name (short)
- Included codes
- Boundary note (what is excluded)
- Evidence IDs supporting the theme
Output must be a table-like JSON with explicit uncertainty markers.

## SI-5.4 Audit-trail fields

Table S3. Audit-trail field set for bounded LLM assistance

| Field | Description | Example value |
| --- | --- | --- |
| run_id | Unique identifier for one model-assisted run | LLM_RUN_2026-03-20_01 |
| prompt_template | Template used (A/B/...) and version | Template A v1.0 |
| evidence_id | Identifier for excerpt (de-identified) | OE-03 |
| input_excerpt | De-identified text passed to model (minimum necessary) | Add more listening audios. |
| model_output_json | Raw structured output saved for audit | {...} |
| uncertainty_flag | Model-provided uncertainty marker | LOW |
| researcher_action | Accept / revise / reject | Revise |
| researcher_rationale | Brief justification for action | Merged with listening-resource subcode |
| final_code_or_theme | Researcher-confirmed code/theme after adjudication | Insufficient listening/input resources |
| timestamp | Date/time of the run | 2026-03-20T10:30Z |

# SI-6. Joint Evidence-to-Decision Display

Table S4 expands the manuscript’s joint evidence-to-decision logic by adding (i) the corresponding CIPP dimension, and (ii) concrete examples of how a revision priority could be operationalized as a classroom or governance practice in a production-constrained setting.

Table S4. Expanded joint evidence-to-decision display with CIPP alignment and concrete examples.

| CIPP dimension | Evidence pattern | Interpretation | Revision priority | Concrete operational example(s) |
| --- | --- | --- | --- | --- |
| Context | High frequency of scenarios and collaborative events; speaking/listening prioritized | Need-task alignment drives perceived relevance and usability | Use collaborative events as instructional backbone | Build reusable frames for asking/clarifying/confirming/reporting; include brief politeness routines and repair strategies. |
| Context/Input | Strong demand for terminology domains (office + industry + etiquette) | General communication and domain terminology should develop together | Layer terminology by role/process | Create department-tagged vocabulary lists with sentence frames; short role cards (production/quality/logistics) for in-class role-play. |
| Input/Process | Pacing rated lowest among Likert items; absence disrupts continuity | Recoverability is a design variable, not only logistics | Build recoverability into course design | Unit learning packs (PDF/mini-lessons), 10–15 minute catch-up tasks; replay audio; peer ‘buddy’ check-ins after missed sessions. |
| Process | Preference for group collaboration and gamified learning; requests for learning community | Low-risk rehearsal and peer support reduce speaking cost | Expand structured interaction opportunities | Rotating pair tasks; weekly micro-dialogue challenges; optional chat group for sharing scripts and Q&A (moderated). |
| Product (transfer-related indicators) | Confidence gains and desire for real-use opportunities; limited direct transfer evidence | Indicators suggest plausibility of later use if opportunities are protected | Add follow-up measures closer to workplace use | Anonymous workplace micro-task checklist; short delayed follow-up survey; optional peer/supervisor note on safe micro-interactions (non-punitive). |
